# The Assessment of Burden of Chronic Conditions (ABCC-) tool: A valid and reliable tool for hip, knee, hand, wrist, foot and ankle osteoarthritis

**DOI:** 10.1016/j.ocarto.2025.100623

**Published:** 2025-05-21

**Authors:** V.H.J. Debie, T.A.E.J. Boymans, A.H.M. Gidding-Slok, O.C.P. van Schayck, R.P.G. Ottenheijm

**Affiliations:** a Department of Family Medicine, Care and Public Health Research Institute (CAPHRI), Maastricht University, Maastricht, the Netherlands; b Department of Orthopedic Surgery, Maastricht University Medical Center, Maastricht, the Netherlands

**Keywords:** ABCC-Tool, Osteoarthritis, Validity, Reliability

## Abstract

**Objective:**

The Assessment of Burden of Chronic Conditions (ABCC-) tool was developed to provide a comprehensive, holistic evaluation of disease burden on physical, emotional and social level in patients with one or more chronic conditions. This study aimed to assess the validity and reliability of the recently developed module for the ABCC-tool for osteoarthritis (OA).

**Design:**

A cross-sectional questionnaire-based study was conducted among patients with hip, knee, hand, wrist, foot and ankle OA. Validity was assessed as (1) the convergent validity with three OA-joint-specific questionnaires measuring pain and stiffness amongst others; (2) the discriminant validity with radiographic Kellgren-Lawrence (KL) scores; and (3) known-groups validity (groups: number of affected joints, and the presence/absence of anxiety, depression, kinesiophobia, and pain catastrophizing). Reliability was assessed as the (1) internal consistency; and (2) test-retest with a two-week interval.

**Results:**

409 OA patients were included. For all joints its convergent validity was according to the hypothesis, i.e. r ​≥ ​0.7 (hip OA r ​= ​-0.73; knee OA r ​= ​-0.75; hand and wrist OA r ​= ​0.74; foot and ankle OA r ​= ​-0.41). As expected according to the hypothesis, no correlation between burden of OA and KL-scores was found for the discriminant validity. The ABCC-tool was able to distinguish all predefined known-groups (all *p* ​< ​0.05). The ABCC-tool demonstrated good internal consistency across all domains and joints (total scale in all joints ≥0.87). Test-retest reliability was high (all ICC ≥0.81).

**Conclusion:**

The ABCC-tool is a valid and reliable tool for assessing the multidimensional burden of OA across different joints.

## 1 Introduction

Despite extensive efforts to develop disease-modifying osteoarthritis (OA) drugs (DMOADs) to halt or slow OA progression, no truly effective medication has been found yet [1]. As a result, over 1.5 million people (9%) in the Netherlands still suffer daily from pain, joint stiffness, and other OA-related symptoms that limit their functioning and reduce quality of life (QoL) [2,3]. Their current treatment focus on symptom management but fail to address the underlying causes of OA [2].

To optimize symptom management and improve QoL, it is paramount that the healthcare professional and patient have a comprehensive understanding of the patient's physical health, emotional well-being and social experiences [4]. Therefore, OA care is shifting to a more person-centered approach, assessing overall health rather than just physical symptoms [5]. To facilitate this person-centered approach, a variety of OA or other health-related questionnaires are used to assess these aspects of health, for example the Western Ontario and McMaster Universities Osteoarthritis Index (WOMAC) for lower extremities and the Disability of the Arm, Shoulder and Hand Questionnaire (DASH) for upper extremities [[6], [7], [8], [9]]. However, existing questionnaires are mostly joint-specific, overlooking other aspects of OA or related conditions, or tend to concentrate solely on physical symptoms or QoL [10]. Furthermore, these questionnaires do not include a summary, for example in the form of a visual, of the questionnaire results. Portraying these results in a visual way helps patients and healthcare providers to overview and understand the experienced burden and could be a helpful support in the conversation during the consultation. This approach could enable greater patient engagement and more autonomy in the care process, which is and is crucial for a person-centered approach. Therefore, a new assessment tool is needed which includes a visual representation.

A previously developed and validated patient reported outcome measure designed to assess this overall health status is the Assessment of Burden of Chronic Conditions (ABCC-) tool. This tool has been developed and validated for asthma, chronic heart failure, COPD, diabetes type 2, and post-COVID. Recently, we developed a module for OA in the ABCC-tool and assessed the content validity of it, ensuring that the content is relevant for addressing the needs of patients with OA [4,[12], [13], [14], [15]].

This tool adopts a holistic approach, assessing patients' experiences across multiple dimensions of health, including symptom severity, functional status, and QoL based on self-reported answers. This allows a comprehensive understanding of the patient's health status [10,12,16]. The ABCC-tool consists of a set of generic questions, applicable for patients with all kinds of chronic conditions, including questions regarding lifestyle. To tailor the patient reported outcome measure to the specific (combination of) chronic condition(s), one or more condition-specific modules can be added to these generic questions, as for each chronic condition in the ABCC-tool a separate module is created. This modular approach allows for individualized assessments, especially in cases of comorbidities [12].

**Fig. 1:**
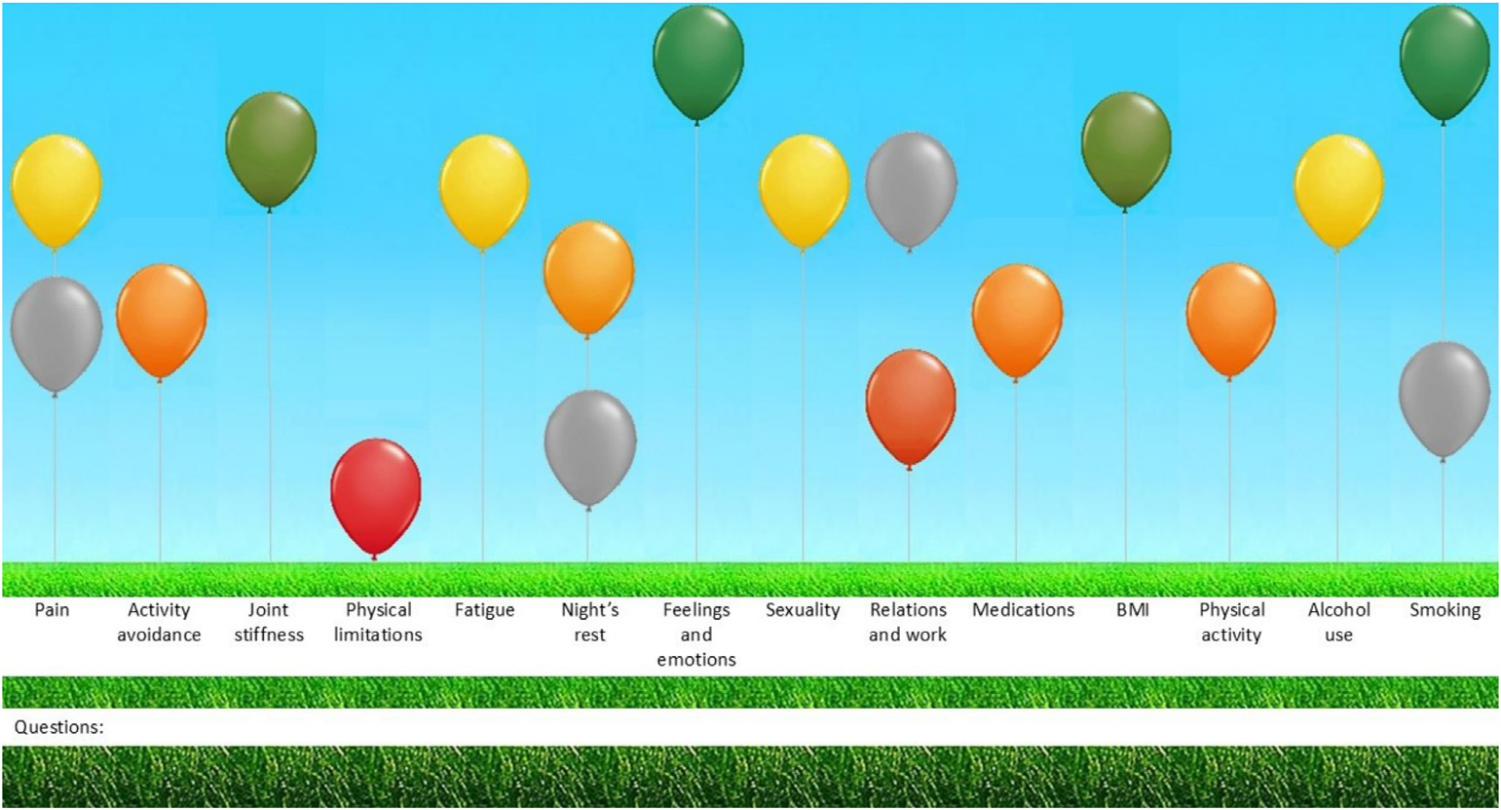
The ABCC-tool visually illustrates the health status of individuals with green-to-red shaded and grey balloons. The more red and lower the balloon, the higher the burden of disease, while greener and higher balloons indicate lower burden of disease. The grey balloons show improvement or deterioration with the previous time. This illustration is an example of a patient with OA. The left three (green/yellow) balloons present condition-specific domains, while the remaining eleven balloons correspond to inquiries from the generic and lifestyle module. (For interpretation of the references to color in this figure legend, the reader is referred to the Web version of this article.)

Results are displayed in a balloon diagram, ranging from green to red and grey balloons (see Fig. 1). This visual supports discussion between patient and healthcare professional but is not intended to be used for diagnostic purposes. Treatment advice for each domain becomes visible by clicking on the corresponding balloon, fostering shared decision-making and personalized treatment goals [[10], [11], [12]]. Shared decision-making enhances treatment by involving patients, who are experts in managing their OA. Self-management programs have been shown to reduce pain, disabilities, and ambulatory visits [17]. Moreover, self-management plays a key role in enhancing patient activation. The ABCC-tool has also been proved to boost patient activation and perceived quality of care in a large clustered, clustered, two-armed, quasi-experimental trial [18].

Before implementing the newly developed module for OA in healthcare practices, it needs to be validated first. Therefore, this study aims to evaluate the construct validity of the total ABCC-scale for OA and its domains by assessing the convergent, discriminant and known-groups validity, and the reliability as the internal consistency and the test-retest.

## 2 Methods

### 2.1 Study design

A cross-sectional questionnaire study was conducted in the Netherlands between December 2023 and June 2024, using the Consensus-Based Standards for the Selection of Health Measurement Instruments (COSMIN) guidelines [19]. This study was approved by the Medical Ethics Committee of Zuyderland, Heerlen (METCZ20210040). Patients provided digital informed consent before participation.

### 2.2 Study population

Patients with OA in the hip, knee, hand, wrist, foot, or ankle were included due to the high prevalence in these joints [20]. Those with OA limited to the shoulder, elbow, back, or neck were excluded: shoulder OA often results from other conditions, elbow OA is rare, and back/neck OA lacks clear diagnostic criteria [[21], [22], [23]]. Participants had to be at least 18 years old and able to read and understand Dutch. Pregnant individuals were excluded due to potential joint instability from hormonal changes [24].

Patients were recruited via three different ways [1]: by a specialized GP in musculoskeletal disorders and orthopedic surgeons, in a primary care orthopedic clinic or hospital, respectively [2]; patients and family members of these patients in the waiting room of the orthopedic department in a hospital; and [3] via the newsletter of ReumaNederland. ReumaNederland is a Dutch health fund and patient advocacy organization committed to supporting individuals with a range of rheumatic diseases, including OA.

No clear rules exist for sample size calculation in validation studies. However, it is advised to maintain a participant-to-item ratio ranging from 2 to 20 participants per item. With a participant-to-item ratio of 8, the study required at least 112 participants per joint for the 14-item scale [25].

### 2.3 Data collection

Both questionnaires and medical imaging of the hip and knee were used in this study. In addition to the ABCC-tool for OA, patients filled in baseline characteristics, one or two joint-specific OA questionnaires and three psychosocial questionnaires. All questionnaires were provided in Dutch. Questionnaire scores and missing data were handled as intended by the original questionnaires. Patients who did not respond were sent two reminders, after four and seven days, respectively.

#### 2.3.1 Questionnaires

##### 2.3.1.1 ABCC-tool for OA

The ABCC-tool is a Dutch self-administered questionnaire with condition-specific modules additionally to the eleven generic and five lifestyle questions. The generic part, relevant for anyone with the included chronic conditions in the ABCC-tool, consists of seven domains: physical limitations (3 items), feelings and emotions (2 items), relations and work (2 items), fatigue (1 item), night's rest (1 item), medication (1 item), and sexuality (1 item). The scale of the ABCC-tool has already been found to be a valid and reliable measurement tool for other chronic conditions, such as COPD and diabetes mellitus type 2 [4,10,12,16]. The condition-specific module for OA consists of three domains: pain (1 item), joint stiffness (1 item), and activity avoidance (1 item). Its content validity has been assessed as good [14]. The generic and OA items are scored with a 7-point Likert scale (0 ​= ​no burden of disease, 6 ​= ​high burden of disease). The calculation of the total and domain scores, and missing items are explained by Claessens et al., 2023 [10]. The full ABCC-scale (Dutch version, and an unvalidated English translation for the purpose of this paper) is presented in Appendix 1.

Based on the affected joints included in this study, ranked from highest to lowest burden, patients completed either one or two of the following joint-specific questionnaires. In case of more than one affected joint, the top two joint-specific questionnaires were filled in. Notably, if the knee and hip were ranked first and second, a third joint-questionnaire was also completed, as both hip and knee assessments were integrated into the same questionnaire. E.g. in case of hip, knee and hand or wrist OA, patients did not only complete the WOMAC, which is a combined questionnaire for hip and knee OA, but also the DASH.

##### 2.3.1.2 DASH

Patients with hand or wrist OA filled in the DASH. The DASH measures disabilities of the arm, shoulder and hand with 30 questions based on three domains: physical function (21 items), symptoms (5 items), and psychosocial factors (4 items). The questionnaire is answered on a 5-point Likert scale. The Dutch version of the DASH showed to be valid and reliable in patients with upper limb disorders [9].

##### 2.3.1.3 FAOS

The Foot and Ankle Outcome Score (FAOS) measures foot and ankle disabilities in patients with OA. The 42-item questionnaire measures on a 5-point Likert scale, and consists of five domains: symptoms (2 items about joint stiffness, 5 items about other symptoms), pain (9 items), functioning in daily life (17 items), functioning in sport and leisure activities (5 items), and QoL (4 items). Domain scores range from 0 to 100, where 0 indicates high burden and 100 no burden of disease. Validity and reliability were assessed with the Dutch version in patients with various foot and ankle complaints, and was sufficient [26].

##### 2.3.1.4 WOMAC

The WOMAC is a self-administered questionnaire measuring knee and/or hip OA. It is divided into three subscales: pain (5 items), joint stiffness (2 items) and physical functioning (17 items). The 24 questions have to be answered on a 5-point Likert scale. Domain scores range from 0 to 100: the higher the score, the lower the burden of disease. Clinimetric properties of the WOMAC were good in Dutch Patients with OA [6,27].

The following three questionnaires measured psychosocial characteristics, and were filled in by all patients regardless the affected joint(s).

##### 2.3.1.5 HADS

The Hospital Anxiety and Depression Scale (HADS) is originally developed to screen for anxiety and depression in medical outpatient clinics. The questionnaire consists of 14-items, divided into two subscales of seven anxiety and seven depression questions, to be answered on a 4-point Likert scale. Per subscale, a score of ≥8 is considered as a depression or anxiety. Total scores per subscale are 21. The Hospital Anxiety and Depression Scale is well validated in different languages, including Dutch, and different populations, such as primary care patients, somatic patients and general population [28].

##### 2.3.1.6 PCS

The Pain Catastrophizing Scale (PCS) is a valid and reliable self-administered questionnaire measuring pain catastrophizing. The PCS contains 13 statements about feelings and thoughts that might come up with pain. These statements are divided into three subscales: rumination (4 items), helplessness (6 items) and magnification (3 items). The statements have to be answered from scores 0 (never) to 4 (always), leading to a maximum score of 52. The threshold for pain catastrophizing is ​≥ ​30. The PCS questionnaire was found to be valid in patients with chronic pain [29].

##### 2.3.1.7 TSK

The Tempa Scale for Kinesiophobia (TSK) is a questionnaire to assess beliefs about bodily damage arising from physical activity. The TSK is originally developed for chronic back pain patients, and is also useable for patients with OA. We used the short version of 11 items, which is recommended if multiple outcome measures are used to get a general biopsychosocial assessment of the patient. The TSK consist of 5 items with a somatic focus and 6 items about activity avoidance. The maximum score is 44, and its threshold for kinesiophobia is ​≥ ​24. The TSK showed good psychometric properties [[30], [31], [32]].

#### 2.3.2 Imaging

For knee and hip OA, available radiographs of the affected hip(s) and/or knee(s) were classified according to the Kellgren Lawrence classification (KL-classification) to assess OA severity, ranging from 0 to 4 (0 ​= ​none; 1 ​= ​doubtful; 2 ​= ​minimal; 3 ​= ​moderate; 4 ​= ​severe). This classification has been commonly used in OA research assessing radiographs of the hip and knee [33]. Radiographs were made in a supine position in the sagittal and coronal plane.

### 2.4 Data analysis

Patients were excluded from the analysis if the ABCC-tool and at least one OA-questionnaire were not fully completed. All statistical analysis were performed with IBM SPSS version 28.0 (IBM Corp, Armonk, NY, USA). Significance level was *p* ​< ​0.05.

#### 2.4.1 Construct validity

Construct validity is defined as ‘the degree to which the content of an instrument is an adequate reflection of the construct to be measured’ [34]. To assess construct validity, the ABCC-scale was assessed for convergent validity, discriminant validity and known-groups validity.

To assess convergent validity, it was hypothesized that the OA joint-specific questionnaires will strongly correlate with the ABCC-scale. In an ideal situation, we would compare the ABCC-scale with a gold standard [35]. However, no gold standard exists for OA. Therefore, we determined the Pearson correlation coefficient between the ABCC-scale and the WOMAC for hip and knee OA, the DASH for hand and wrist OA, and the FAOS for foot and ankle OA. A strong Pearson's correlation was expected for the total scales: r ​≥ ​0.70 for the DASH and r ​≤ ​-0.70 for the WOMAC and FAOS. For the domains a correlation of r ​≥ ​0.30 for the DASH and r ​≤ ​-0.30 for the WOMAC and FAOS was anticipated, as for single-item domains or domains with only limited items, only a moderate correlation was expected [10,36]. A negative correlation for the WOMAC and FAOS was expected as these questionnaires' scores are in opposite direction than the ABCC-scale. The construct validity received a positive assessment when at least 75 ​% of the outcomes aligned with the predefined hypotheses within (sub)groups containing at least 50 patients [37]. An additional analysis was performed for each joint ranked as having the highest burden of OA, as it is likely that patients completed the ABCC-tool based on the joint causing the most burden.

To assess the discriminant validity, we expected a moderate to low correlation between pain and the severity of radiographic knee and hip OA, as previous research showed that there is no strong relationship between patients’ symptoms and radiographic OA [38]. The expected Spearman correlation coefficient would be ​≤ ​0.50 [36]. Only patients seen in a clinical context were considered for this analysis. Additionally, the orthopedic surgeon must have had an established therapeutic relationship with the patient to ensure compliance with privacy regulations.

To assess known-group validity, we predefined five categories. Per category, patients were divided into two groups: number of affected joints (1 vs. ≥2); presence/absence of anxiety (HADS anxiety subscale score <8 vs. ≥8); presence/absence of depression (HADS depression subscale score <8 vs. ≥8); presence/absence of pain catastrophizing (PCS <30 vs. ≥30); and presence/absence of kinesiophobia (TSK <24 vs. ≥24). Per category, the ABCC domain scores were compared between the two groups. After checking for normality with the Shapiro-Wilk test, differences between groups were analyzed by an independent-sample T-test or Mann-Whitney *U* test, with a p-value of <0.05 considered as statistically significant. The hypotheses regarding known-groups are based on literature and expert opinion, and presented in Appendix 2.

#### 2.4.2 Reliability

In this study, reliability was assessed as internal consistency and test-retest. To determine internal consistency, Cronbach's alpha was calculated for the total ABCC-scale and the multi-item domains. In general, having more items increases Cronbach's alpha. An acceptable Cronbach's alpha is ​≥ ​0.90 for the total scale, and ≥0.70 for subscales [10,34,39].

Test-retest reliability was measured as the Intraclass Correlation Coefficient (ICC), with two weeks in between. The first set of questionnaires was taken at baseline, and two weeks later, patients filled in only the ABCC-scale once more. In addition, we asked patients if their pain had increased, decreased or remained the same. Those patients reporting changes in pain were excluded from the test-retest analysis. An acceptable reliability (ICC ≥0.70) between the assessments was hypothesized [35,40].

## 3 Results

### 3.1 Baseline characteristics

A total of 534 patients with OA were enrolled in this study, of which 409 patients were included in the analysis. 137 patients had 1 affected joint, 272 patients had 2 or more affected joints. 140 patients reported hip OA and 228 knee OA, resulting in 249 completed WOMAC's. 279 patients had hand or wrist OA and 261 of them completed the DASH. Foot or ankle OA was reported by 166 patients, with 124 of them completing the FAOS. 162 patients also reported OA at their neck, back, shoulder or elbow. See Table 1 for more characteristic details, and Appendix 3 for the complete flowchart.

**Table 1:** Baseline characteristics.

	Total	Hip OA	Knee OA	Hand or wrist OA	Foot or ankle OA
*n ​=*	*409*^a^	*123*	*203*	*261*	124
Gender (n, (%))	F: 326 (80 ​%) M: 83 (20 ​%)	F: 95 (77 ​%) M: 28 (23 ​%)	F: 160 (79 ​%) M: 43 (21 ​%)	F: 221 (85 ​%) M: 40 (15 ​%)	F: 99 (80 ​%) M: 25 (20 ​%)
Age (years; median, (IQR))	63.0 (57.0–69.0)	64.0 (58.0–70.0)	64.0 (57.0–70.0)	64.0 (58.0–69.0)	63.0 (57.0–68.8)
Time since OA diagnosis (years; median, (IQR))	7.0 (3.0–14.0)	8.0 (3.9–15.00)	8.0 (3.0–15)	8.0 (4.0–15.0)	8.5 (4.3–15.0)
Other chronic conditions (n, (%))	245 (60 ​%)	79 (64 ​%)	129 (64 ​%)	159 (61 ​%)	68 (55 ​%)

a Patients may have more than one affected joint.

### 3.2 Validity

#### 3.2.1 Convergent validity

The correlation between the total scores of the ABCC-scale and WOMAC was r ​= ​−0.75, exceeding the predefined threshold. When splitting the WOMAC scores per joint, patients with knee OA score a correlation of −0.75 and those with hip OA −0.73 between the WOMAC and ABCC-scale. The correlation between the total score of the DASH and ABCC-scale was 0.74, and the FAOS had a correlation of −0.41 with the ABCC-scale. Table 2 provides a total overview of all correlations per scale and their domains.

**Table 2:** Convergent and discriminant validity.

		All patients with OA at this joint		All patients with OA at this joint AND ranked this joint with the highest burden of disease	
		r	*p*	r	*p*
**Hip OA**					
***Convergent validity***					
	*n* ​=	*123*		*42*	
**WOMAC**	**ABCC-scale**				
Total scale	Total scale	−0.73	<0.001	−0.76	<0.001
Pain	Pain	−0.71	<0.001	−0.83	<0.001
Joint stiffness	Joint stiffness	−0.54	<0.001	−0.56	<0.001
**TSK**					
Total scale	Activity avoidance	0.48	<0.001	0.53	<0.001
***Discriminant validity***					
	*n* ​=	*17*			
**Radiographs**	**ABCC-scale**				
KL-score	Pain	−0.08	0.772		
KL-score	Joint stiffness	−0.06	0.816		
KL-score	Activity avoidance	0.08	0.753		
**Knee OA**					
***Convergent validity***					
	*n* ​=	*203*		*94*	
**WOMAC**	**ABCC-scale**				
Total scale	Total scale	−0.75	<0.001	−0.78	<0.001
Pain	Pain	−0.67	<0.001	−0.69	<0.001
Joint stiffness	Joint stiffness	−0.56	<0.001	−0.49	<0.001
**TSK**					
Total scale	Activity avoidance	0.38	<0.001	0.25	0.017
***Discriminant validity***					
	*n* ​=	*26*			
**Radiographs**	**ABCC-scale**				
KL-score	Pain	−0.04	0.851		
KL-score	Joint stiffness	0.02	0.929		
KL-score	Activity avoidance	0.09	0.666		
**Hand and wrist OA**					
***Convergent validity***					
	*n* ​=	*261*		*156*	
**DASH**	**ABCC-scale**				
Total scale	Total scale	0.74	<0.001	0.76	<0.001
Symptoms	Pain ​+ ​joint stiffness	0.60	<0.001	0.66	<0.001
**TSK**					
Total scale	Activity avoidance	0.36	<0.001	0.46	<0.001
**Foot and ankle OA**					
***Convergent validity***					
	*n* ​=	*124*		*55*	
**FAOS**	**ABCC-scale**				
Total scale	Total scale	−0.41	<0.001	−0.60	<0.001
Pain	Pain	−0.42	<0.001	−0.66	<0.001
Joint stiffness	Joint stiffness	−0.31	<0.001	−0.42	0.002
**TSK**					
Total scale	Activity avoidance	0.30	<0.001	0.31	0.021

#### 3.2.2 Discriminant validity

KL scores were obtained for 17 patients with hip OA and 26 patients with knee OA. Correlations between KL scores and OA-related questions on the ABCC-scale ranged from −0.08 to 0.08 for hip OA and from −0.04 to 0.09 for knee OA, as presented in Table 2.

#### 3.2.3 Known-groups

Patients with OA with two or more affected joints, and the presence of anxiety, depression, kinesiophobia, or pain catastrophizing scored significant higher on all domains of the ABCC-scale (all *p* ​< ​0.05) than patients with OA with one affected joint, or the absence of anxiety, depression, kinesiophobia, and pain catastrophizing, respectively. See Appendix 4 for more details.

### 3.3 Reliability

#### 3.3.1 Internal consistency

The Cronbach's alpha of the total ABCC-scale was 0.90. The internal consistency of patients with hip OA on the total ABCC-scale was 0.87, for knee OA 0.88, for hand or wrist OA 0.90, and for foot or ankle OA 0.88. See Table 3 for the results per domain and per joint.

**Table 3:** Reliability.

	Total	Hip OA	Knee OA	Hand and wrist OA	Foot and ankle OA
***Internal consistency (Cronbach's alpha)***					
*n* ​=	*409*	*123*	*203*	*261*	*124*
Total scale	0.90	0.87	0.88	0.90	0.88
OA specific questions	0.72	0.68	0.72	0.73	0.72
Physical limitations	0.84	0.85	0.83	0.85	0.80
Feelings and emotion	0.69	0.59	0.62	0.71	0.76
Relations and work	0.71	0.77	0.65	0.72	0.66
***Test-retest (Intraclass correlation coefficient)***					
*n* ​=	*227*	*87*	*120*	*156*	*99*
Total scale	0.83	0.82	0.81	0.82	0.81

#### 3.3.2 Test-retest

Between T0 and T1, the mean interval duration was 15.8 days. 227 patients reported no change in burden of disease, and scored an ICC of 0.83 for the total scale. All separate joints scored an ICC between 0.81 and 0.82 on the total ABCC-scale. More details are presented in Table 3.

## 4 Discussion

This study assessed the ABCC-tool as a reliable and valid instrument for assessing the burden of disease in patients with hip, knee, hand, wrist, foot and ankle OA. Our primary hypothesis was that the ABCC-tool would show a strong correlation with the OA-specific questionnaires. As expected, the ABCC-scale showed indeed strong correlations with both the WOMAC and DASH (r ​= ​−0.75, and r ​= ​0.74, respectively). The correlation between the FAOS and ABCC-scale was −0.41, which is lower than was hypothesized. However, given that the FAOS focuses on condition-specific outcomes for foot and ankle OA while the ABCC-tool assesses more general health outcomes, this lower correlation is acceptable for considering the ABCC-tool valid for foot and ankle OA. Furthermore, more than 75% of the subdomains scored according to the hypothesis of r ​≤ ​−0.30 and r ​≥ ​0.30.

At the time the ABCC-tool will be used in clinical practice, it is expected that the patient and healthcare professional will focus on the most burdensome joint. Therefore, we performed an additional analysis for each joint if it was ranked as the highest burden. This revealed an even stronger correlation between the total scores of the OA-specific questionnaires and the ABCC-tool, as well as nearly all subdomains for all included joints.

As hypothesized, no correlation was found between the OA domains and the KL-scores of the knee and hip pain, joint stiffness and activity avoidance (all r between −0.08 and 0.09). This is in line with previous research, where no correlation between self-reported outcomes and radiographic outcomes was found [41]. Another study found a weak correlation of 0.23–0.26 between self-reported and radiographic OA outcomes [42]. Radiographic imaging primarily visualizes bone structures, yet other factors such as synovitis, muscles and ligaments, which are not captured on these images, are also key contributors to OA pathology and thus symptoms [41].

Furthermore, we predefined known-groups and hypotheses before the study, and the ABCC-scale successfully distinguished these groups across all domains as expected. Interestingly, additional significant differences emerged across other domains, offering unexpected insights beyond our original hypothesis.

This study shows a strong internal consistency for both the total ABCC-scale and its domains. For example, the domain “feelings and emotions” scored lower as expected among hip and knee patients. This is likely due to the less acute and life-threatening nature of OA compared to other chronic conditions in the ABCC-tool, resulting in fewer severe symptoms and daily life restrictions. For COPD, asthma and DM2, the internal consistency of the domain “feelings and emotions” was 0.77, 0.74 and 0.76, respectively [10]. Given these values and nature of the condition, this domain will not be split in two separate domains in the ABCC-tool as it is intended to be applicable across all included chronic conditions. The internal consistency for hip, knee, and foot and ankle OA ranges from 0.87 to 0.88. Although this is slightly below the predefined threshold of 0.90, these values still fall within the generally accepted range for an acceptable internal consistency (≥0.70) [39]. The 0.02–0.03 deviation from the hypothesized value can be considered marginal from a statistical perspective and is unlikely to have a meaningful impact on the reliability of the measurement instruments. Furthermore, the test-retest reliability assessment was good for the total group (ICC ​= ​0.83) and for each joint (all joints ICC between 0.81 and 0.82).

The main purpose of the ABCC-tool is to visualize the burden of disease and support the conversation during a consultation for patients with one or more chronic conditions, rather than to serve diagnostic purposes. Additionally, the tool was developed for use in clinical settings rather than for scientific research. The questionnaire within the tool is intentionally kept as brief as possible, with the expectation that healthcare professionals will explore topics further through conversation with the patient. In contrast, the questionnaires used in this study (e.g., the WOMAC) are designed for both clinical practice and research purposes [6]. These instruments are more extensive and include a greater number of questions per domain.

### 4.1 Strengths and limitations

This study has several strengths and limitations. First, our study had a large sample size, encompassing 409 patients with OA, with a significant majority being female (80%). This sample size exceeded the minimal number of patients of 112 per joint, with 123 times hip OA, 203 knee OA, 261 hand and wrist OA, and 124 foot and ankle OA. The gender distribution roughly aligns with the general Dutch OA population, where the majority of the patients is female (65%), ensuring that our study population accurately reflects the real-world demographics.

Additionally, by recruiting patients through GPs, orthopedic surgeons, waiting rooms, and online, we were able to capture a heterogeneous patient population. This included individuals who had consulted healthcare professionals and received an official OA diagnosis, with or without undergoing an intervention, as well as those who self-reported OA. For patients who self-reported OA, particularly those recruited via ReumaNederland or from the waiting room, it was not possible to verify their OA diagnosis. This was also the case for patients who visited a healthcare professional for a specific joint but self-reported OA in other joints. Despite this limitation, the diverse recruitment strategy allowed us to analyze outcomes across a wide range of patient experiences and clinical scenarios, and thereby enhancing the generalizability of our findings.

We chose to have participants complete a maximum of two OA-specific questionnaires, which could be a limitation of this study. For participants who had to complete the two longest OA-questionnaires, the DASH with 30 items and the FAOS with 42 items, the total number of questions exceeded 200. Adding a third OA-specific questionnaire would have imposed an excessive burden on participants. Moreover, it is uncertain whether meaningful data could be obtained for more than two joints using a questionnaire format. Therefore, participants were limited to a maximum of two OA-specific questionnaires, targeting on the two joints with the highest reported burden of disease.

A final limitation is the lack of a defined threshold for the 11-item TSK in the literature. While the 17-item version uses 55% of the total score (maximum score: 68, threshold: 37) [43], no such cutoff exists for the shorter version, which is recommended for broader biopsychosocial assessments [31]. Therefore, we set the threshold at 24, reflecting 55% of its maximum score of 44.

## 5 Conclusion

In conclusion, the ABCC-tool for OA is a self-administered questionnaire that is not joint-specific and includes all aspects of OA burden, i.e. physical, emotional and social burden. The purpose of this tool is to support the conversation between the patient and healthcare professional. This study showed that the ABCC-tool is valid and reliable for hip, knee, hand, wrist, foot and ankle OA.

## Author contribution

VD, AGS, RO, OvS and TB had a substantial contribution to the validation of the OA module for the ABCC-tool. Data analysis was done by VD, and discussed with all authors. AGS, RO, TB and OvS had a supervisory role during the process. The first manuscript was written by VD, which was critically evaluated by all authors. The final version was read and approved by all authors as well. All authors agreed on the content of the work related to accuracy and integrity.

## Role of the funding source

This study was funded by 10.13039/501100006315ReumaNederland (NSP22-1-303). The funding party had no role in study design, analysis, interpretation or manuscript writing.

## Declaration of competing interest

There are no conflicts of interest.

## Footnotes

Appendix A Supplementary data to this article can be found online at https://doi.org/10.1016/j.ocarto.2025.100623.
