# Supplementary material for: The Assessment of Burden of Chronic Conditions (ABCC-) tool: A valid and reliable tool for hip, knee, hand, wrist, foot and ankle osteoarthritis
Source: Osteoarthr Cartil Open. 2025 May 21;7(3):100623. doi: 10.1016/j.ocarto.2025.100623 (PMC12164024; doi:10.1016/j.ocarto.2025.100623)
Supplement: Multimedia component 1 [file mmc1.docx]

# Appendix 1: Assessment of Burden of Chronic Conditions (ABCC-) tool for OA

## English version

*(Note: this is an unvalidated translation from Dutch to English specifically for this paper)*

| Dear Sir/Madam.  With this questionnaire, we would like to get an impression of how you are doing. During consultation with your healthcare provider, you can talk about the topics that are important to you.  These questions are related to the chronic condition(s) for which you are visiting the healthcare provider. | | | | | | | | |
| --- | --- | --- | --- | --- | --- | --- | --- | --- |
| **Generic questions** | | | | | | | | |
| **In the past week, how often ….** | | **0** | **1** | **2** | **3** | **4** | **5** | **6** |
|  |  | **Never** | **Hardly ever** | **A few times** | **Several times** | **Many times** | **A great many times** | **Almost all the time** |
| 1 | did you suffer from **fatigue**? | **□** | **□** | **□** | **□** | **□** | **□** | **□** |
| 2 | did you have a poor **night’s rest**? | **□** | **□** | **□** | **□** | **□** | **□** | **□** |
| 3 | did you suffer from sadness, fear, frustration, shame or other **unpleasant feelings**? | **□** | **□** | **□** | **□** | **□** | **□** | **□** |
| 4 | did you experience taking **medication** (e.g. tablets, puffs, insulin) as a burden? | **□** | **□** | **□** | **□** | **□** | **□** | **□** |
| **In the past week, to what extend …** | | **0** | **1** | **2** | **3** | **4** | **5** | **6** |
|  |  | **Not at all** | **Very slightly** | **Slightly** | **Moderate** | **Very** | **Extremely** | **Totally** |
| 5 | were you limited in **strenuous physical** activities (such as climbing stairs, hurrying, doing sports)? | □ | □ | □ | □ | □ | □ | □ |
| 6 | were you limited in **moderate physical** activities (such as walking, housework, carrying things)? | □ | □ | □ | □ | □ | □ | □ |
| 7 | were you limited in **daily activities** at home (such as dressing, washing yourself) | □ | □ | □ | □ | □ | □ | □ |
| 8 | were you limited in your **work or social activities** (short trip., visiting friends and family)? | **□** | **□** | **□** | **□** | **□** | **□** | **□** |
| 9 | had your condition a negative impact on your **relations with others**? | **□** | **□** | **□** | **□** | **□** | **□** | **□** |
| 10 | did you have any difficulty with **intimacy or sexuality**? | **□** | **□** | **□** | **□** | **□** | **□** | **□** |
| 11 | did you **worry about your future**? | **□** | **□** | **□** | **□** | **□** | **□** | **□** |
|  |  |  |  |  |  |  |  |  |
| **OA-specific questions** | | | | | | | | |
| **In the past month, to what extend …** | | **0** | **1** | **2** | **3** | **4** | **5** | **6** |
|  |  | **Not at all** | **Very slightly** | **Slightly** | **Moderate** | **Very** | **Extremely** | **Totally** |
| 12 | did you suffer from **pain**? | **□** | **□** | **□** | **□** | **□** | **□** | **□** |
| 13 | did you **avoid activities** to prevent an increase in pain? | **□** | **□** | **□** | **□** | **□** | **□** | **□** |
| 14 | did you suffer from **joint stiffness** (the feeling that the joint does not move smoothly)? | **□** | **□** | **□** | **□** | **□** | **□** | **□** |
| **Lifestyle questions** | | | | | | | | |
| **The following questions relate to your lifestyle** | | | | | | | | |
| 15 | In the past week, how many days have you had moderately intense **physical exercise** for 30 minutes or more?  E.g. walking or cycling in a fast pace.  It may also be a minimum of 3x10 minutes. | □ 0 days  □ 1-2 days  □ 3-4 days  □ 5 days or more | | | | | | |
| 16 | How many glasses of **alcohol** did you drink in the past week? | … glasses per week | | | | | | |
| 17 | Do you **smoke** or have you smoked? | □ Yes. In the last week, how many (shag) cigarettes have you smoked on average per day? ___________  □ Previously. Stopped smoking since: ____ (month) / ____ (year)  □ Never | | | | | | |
| 18 | What is your **weight**? | ………… kg | | | | | | |
| 19 | What is your **height**? | ………… cm | | | | | | |
| 20 | Is there anything else you would like to **discuss** or would like to receive **more information** about? | | | | | | | |
|  | _________________________________________________________________________________________________________________________________________________________________________________________________________________________________________________________________________________ | | | | | | | |

## Dutch version

| Beste meneer/mevrouw.  Met deze vragenlijst willen we samen met u in kaart brengen hoe het met u gaat.  Deze vragen hebben te maken met de chronische aandoening(en) waarvoor u bij de zorgverlener komt. | | | | | | | | |
| --- | --- | --- | --- | --- | --- | --- | --- | --- |
| **Generieke vragen** | | | | | | | | |
| **In de afgelopen week, hoe vaak …** | | **0** | **1** | **2** | **3** | **4** | **5** | **6** |
|  |  | **Nooit** | **Zelden** | **Af en toe** | **Regel-**  **matig** | **Heel vaak** | **Meestal** | **Altijd** |
| 1 | had u last van **vermoeidheid**? | □ | □ | □ | □ | □ | □ | □ |
| 2 | had u een slechte **nachtrust**? | □ | □ | □ | □ | □ | □ | □ |
| 3 | had u last van somberheid, angst, frustratie, schaamte of andere **vervelende gevoelens**? | □ | □ | □ | □ | □ | □ | □ |
| 4 | ervaarde u het gebruik van **medicijnen** (bijv. tabletten, pufjes, insuline) als een last? | □ | □ | □ | □ | □ | □ | □ |
| **In de afgelopen week, in welke mate …** | | **0** | **1** | **2** | **3** | **4** | **5** | **6** |
|  |  | **Helemaal niet** | **Heel weinig** | **Een beetje** | **Tame-lijk** | **Erg** | **Heel erg** | **Volledig** |
| 5 | voelde u zich beperkt in **zware lichamelijke activiteiten** (trap lopen, haasten, sporten)? | □ | □ | □ | □ | □ | □ | □ |
| 6 | voelde u zich beperkt in **matige lichamelijke activiteiten** (wandelen, huishoudelijk werk, boodschappen doen)? | □ | □ | □ | □ | □ | □ | □ |
| 7 | voelde u zich beperkt in **dagelijkse activiteiten** (u zelf aankleden, wassen)? | □ | □ | □ | □ | □ | □ | □ |
| 8 | voelde u zich beperkt in uw **werk of sociale activiteiten** (uitjes, vrienden en familie bezoeken)? | □ | □ | □ | □ | □ | □ | □ |
| 9 | had uw aandoening een negatieve invloed op uw **relatie met anderen**? | □ | □ | □ | □ | □ | □ | □ |
| 10 | had u moeite met **intimiteit of seksualiteit**? | □ | □ | □ | □ | □ | □ | □ |
| 11 | maakte u zich **zorgen over uw toekomst**? | □ | □ | □ | □ | □ | □ | □ |
|  |  |  |  |  |  |  |  |  |
| **Artrose-specifieke vragen** | | | | | | | | |
| **In de afgelopen maand, in welke mate…** | | **0** | **1** | **2** | **3** | **4** | **5** | **6** |
|  |  | **Helemaal niet** | **Heel weinig** | **Een beetje** | **Tame-lijk** | **Erg** | **Heel erg** | **Volledig** |
| 12 | had u last van pijn? | □ | □ | □ | □ | □ | □ | □ |
| 13 | heeft u activiteiten ontweken om pijn of toename van artrose te voorkomen? | □ | □ | □ | □ | □ | □ | □ |
| 14 | heeft u last gehad van gewrichtstijfheid (het gevoel dat uw gewricht minder soepel beweegt)? | □ | □ | □ | □ | □ | □ | □ |
| **Leefstijl vragen** | | | | | | | | |
| **De volgende vragen gaan over uw leefstijl** | | | | | | | | |
| 15 | In de afgelopen week, hoeveel dagen heeft u 30 minuten of meer matig intensieve lichaamsbeweging gehad?  Bijv. stevig doorwandelen of harder fietsen.  Het mogen ook minimaal 3x10 minuten zijn. | □ 0 dagen  □ 1-2 dagen  □ 3-4 dagen  □ 5 dagen of meer | | | | | | |
| 16 | Hoeveel glazen alcohol dronk u in de afgelopen week? | … glazen per week | | | | | | |
| 17 | Rookt u of heeft u gerookt? | □ Ja. Hoeveel (shag)sigaretten heeft u in de afgelopen week gemiddeld per dag gerookt? ___________  □ Vroeger. Gestopt met roken sinds: ___(maand) / ___(jaar)  □ Nooit | | | | | | |
| 18 | Wat is uw gewicht? | ………… kg | | | | | | |
| 19 | Wat is uw lengte? | ………… cm | | | | | | |
| 20 | Is er nog iets wat u wilt bespreken of waar u meer informatie over wilt krijgen? | | | | | | | |
|  | _________________________________________________________________________________________________________________________________________________________________________________________________________________________________________________________________________________ | | | | | | | |
