# Supplementary material for: The Assessment of Burden of Chronic Conditions (ABCC-) tool: A valid and reliable tool for hip, knee, hand, wrist, foot and ankle osteoarthritis
Source: Osteoarthr Cartil Open. 2025 May 21;7(3):100623. doi: 10.1016/j.ocarto.2025.100623 (PMC12164024; doi:10.1016/j.ocarto.2025.100623)
Supplement: Multimedia component 2 [file mmc2.docx]

# Appendix 2: Known-groups hypotheses

| Table 4: Known-groups hypotheses | | | | | |
| --- | --- | --- | --- | --- | --- |
|  | **1 affected joint vs.**  **≥2 affected joints** | **HADS anxiety subscale**  **<8: no anxiety vs.**  **≥8: anxiety** | **HADS depression subscale**  **<8: no depression vs.**  **≥8: depression** | **TSK <24: no kinesiophobia vs.**  **TSK ≥24: kinesiophobia** | **PCS <30: no pain catastrophizing vs.**  **PCS ≥30: pain catastrophizing** |
| **Physical limitations** | OA at ≥2 affected joints affects patients’ physical fitness (40). | A decline in mental health can cause difficulties and impairments in daily activities (41). | A decline in mental health can cause difficulties and impairments in daily activities (41). | Patients with OA with pain-related fear, measured with the TSK. experience lower daily functioning and lower levels of physical activity (29). | A correlation was found between physical disability and catastrophizing (42). |
| **Feelings and emotions** | OA at ≥2 affected joints affects patients’ feelings (40). | People with anxiety have a worse mental health and increased negative thoughts about the future (43, 44). | People with a depression have a worse mental health and increased negative thoughts about the future (43, 44). | People with kinesiophobia have a worse mental health (45). |  |
| **Relations and work** | OA at ≥2 affected joints affects patients’ daily and social activities (40). | Anxiety has a negative effect on work productivity (46). | A depression has a negative effect on work productivity (46, 47). | Patients with OA with pain-related fear, measured with the TSK. experience lower daily functioning and lower levels of physical activity (29). | Pain catastrophizing is highly correlated with depression and anxiety (48). |
| **Fatigue** |  | People with anxiety are more fatigued (43). | People with a depression are more fatigued (43). |  | Work limitations is correlated with  pain catastrophizing (42). |
| **Night’s rest** |  | People with anxiety have sleep difficulties (43). | People with a depression have sleep difficulties (43). |  |  |
| **Medication** |  |  |  |  |  |
| **Sexuality** |  |  |  |  |  |
| **Pain** | Pain levels increase as the number of affected joints increase (49). | Patients with OA with anxiety show more OA severity. higher pain intensity and pain at multiple sites compared to Patients with OA without anxiety (50). | Patients with OA with a depression show more OA severity. higher pain intensity and pain at multiple sites compared to Patients with OA without a depression (50). | Patients with OA with pain-related fear, measured with the TSK, experience more pain intensity, and lower daily functioning (29, 51). |  |
| **Kinesiophobia** |  |  | People with a depression score worse on kinesiophobia (45). |  | Pain is correlated with pain catastrophizing (42, 51). |
| **Joint stiffness** |  |  | A depression could cause more knee stiffness (52). |  | People who catastrophically misinterpret pain have a high chance of avoidance behaviors. such as avoidance of movement and physical activity (53). |
