# Supplementary material for: The Assessment of Burden of Chronic Conditions (ABCC-) tool: A valid and reliable tool for hip, knee, hand, wrist, foot and ankle osteoarthritis
Source: Osteoarthr Cartil Open. 2025 May 21;7(3):100623. doi: 10.1016/j.ocarto.2025.100623 (PMC12164024; doi:10.1016/j.ocarto.2025.100623)
Supplement: Multimedia component 3 [file mmc3.docx]

# Appendix 3: Flowchart


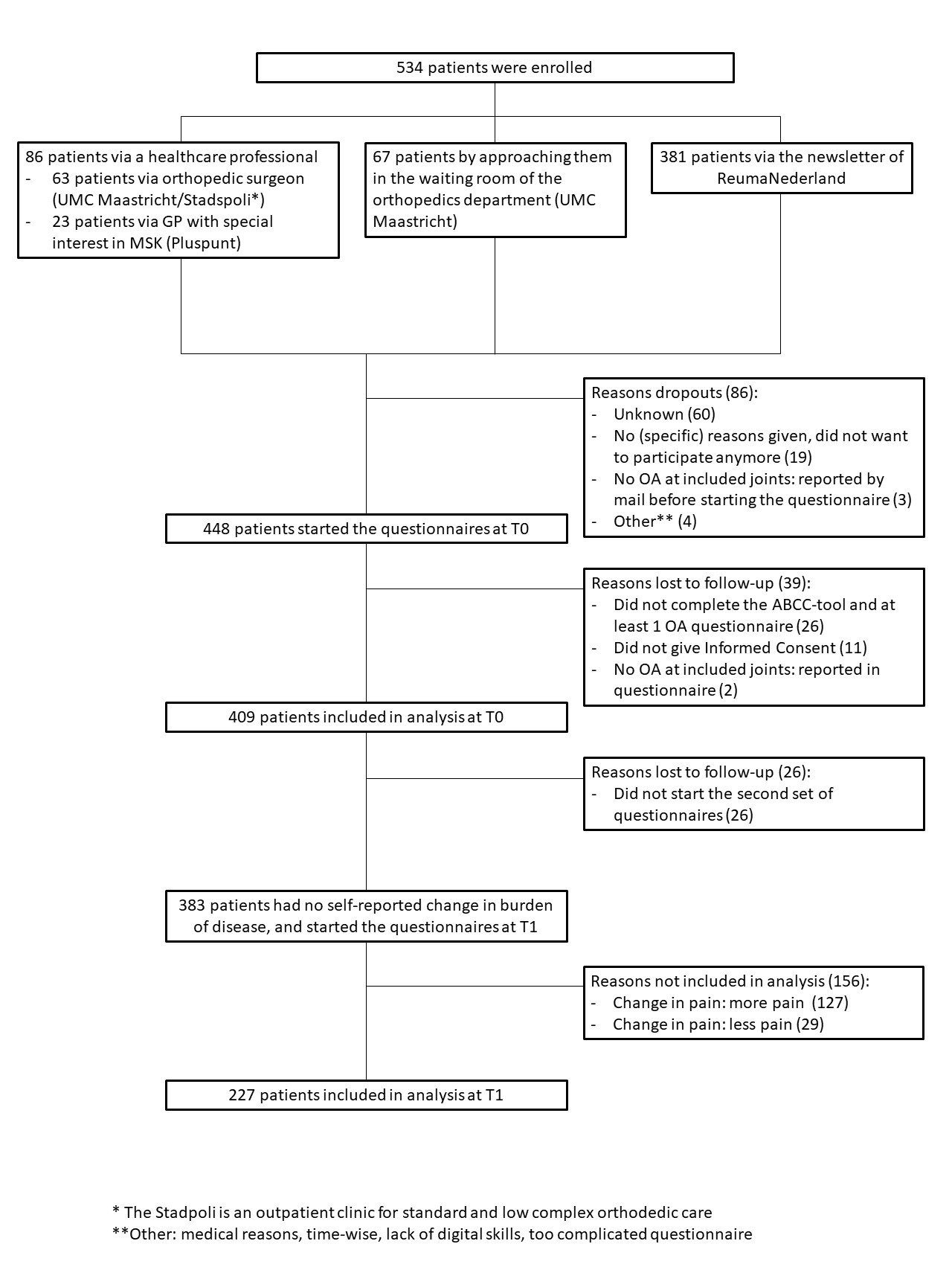


*Figure 2: Flowchart of patient inclusion.
* The Stadspoli is an outpatient clinic for standard and low complex orthopedic care.*

*** Other: medical reasons. time-wise, lack of digital skills, too complicated questionnaire.*
