# Supplementary material for: The Assessment of Burden of Chronic Conditions (ABCC-) tool: A valid and reliable tool for hip, knee, hand, wrist, foot and ankle osteoarthritis
Source: Osteoarthr Cartil Open. 2025 May 21;7(3):100623. doi: 10.1016/j.ocarto.2025.100623 (PMC12164024; doi:10.1016/j.ocarto.2025.100623)
Supplement: Multimedia component 4 [file mmc4.docx]

# Appendix 4: Known-groups results

| Table 5: Known-groups results | | | | | | | | | | | | | | | |
| --- | --- | --- | --- | --- | --- | --- | --- | --- | --- | --- | --- | --- | --- | --- | --- |
|  | **Affected joints** | | | | | **Anxiety** | | | | | **Depression** | | | | |
|  | 1 | | >2 | |  | <8: no anxiety | | ≥8: anxiety | |  | <8: no depression | | ≥8: depression | |  |
| *n =* | *137* | | *272* | |  | 288 | | 117 | |  | *294* | | *111* | |  |
|  | Mean | ±SD | Mean | ±SD | *p* | Mean | ±SD | Mean | ±SD | *p* | Mean | ±SD | Mean | ±SD | *p* |
| **Physical limitations** | 2,36 | 1,26 | 2,93 | 1,24 | <0,001 | 2.55 | 1.26 | 3.18 | 1.17 | <0.001 | 2.44 | 1.22 | 3.50 | 1.05 | <0.001 |
| **Feelings and emotions** | 1,77 | 1,07 | 2,31 | 1,20 | <0,001 | 1.73 | 0.97 | 3.10 | 1.12 | <0.001 | 1.71 | 0.94 | 3.23 | 1.07 | <0.001 |
| **Relations and work** | 1,40 | 1,22 | 1,94 | 1,23 | <0,001 | 1.52 | 1.19 | 2.33 | 1.23 | <0.001 | 1.42 | 1.11 | 2.64 | 1.19 | <0.001 |
| **Fatigue** | 2,76 | 1,45 | 3,61 | 1,46 | <0,001 | 3.12 | 1.53 | 3.84 | 1.33 | <0.001 | 2.99 | 1.46 | 4.21 | 1.25 | <0.001 |
| **Night’s rest** | 2,64 | 1,62 | 3,23 | 1,50 | <0,001 | 2.78 | 1.53 | 3.65 | 1.50 | <0.001 | 2.71 | 1.43 | 3.89 | 1.59 | <0.001 |
| **Medication** | 1,15 | 1,50 | 1,43 | 1,43 | 0,005 | 1.08 | 1.28 | 1.89 | 1.62 | <0.001 | 1.08 | 1.26 | 1.93 | 1.66 | <0.001 |
| **Sexuality** | 1,18 | 1,59 | 1,76 | 1,80 | <0,001 | 1.27 | 1.62 | 2.22 | 1.88 | <0.001 | 1.19 | 1.56 | 2.50 | 1.87 | <0.001 |
| **Pain** | 3,22 | 1,19 | 3,90 | 1,11 | <0,001 | 3.50 | 1.19 | 4.09 | 1.05 | <0.001 | 3.41 | 1.13 | 4.33 | 1.06 | <0.001 |
| **Activity avoidance** | 2,51 | 1,45 | 3,02 | 1,59 | 0,004 | 2.63 | 1.53 | 3.35 | 1.49 | <0.001 | 2.47 | 1.44 | 3.81 | 1.43 | <0.001 |
| **Joint stiffness** | 3,33 | 1,30 | 3,79 | 1,30 | <0,001 | 3.48 | 1.32 | 4.00 | 1.25 | <0.001 | 3.35 | 1.31 | 4.39 | 1.03 | <0.001 |
|  | **Kinesiophobia** | | | | | **Pain catastrophizing** | | | | |  | | | | |
|  | <24: no kinesiophobia | | ≥24: kinesiophobia | |  | <30: no pain catastrophizing | | ≥30: pain catastrophizing | |  |  | |  | |  |
| *n =* | *229* | | *171* | |  | *344* | | *59* | |  |  | |  | |  |
|  | Mean | ±SD | Mean | ±SD | *p* | Mean | ±SD | Mean | ±SD | *p* |  |  |  |  |  |
| **Physical limitations** | 2.52 | 1.30 | 3.03 | 1.15 | <0.001 | 2.61 | 1.25 | 3.50 | 1.10 | <0.001 |  |  |  |  |  |
| **Feelings and emotions** | 1.80 | 1.06 | 2.58 | 1.20 | <0.001 | 1.93 | 1.07 | 3.29 | 1.19 | <0.001 |  |  |  |  |  |
| **Relations and work** | 1.52 | 1.18 | 2.08 | 1.28 | <0.001 | 1.59 | 1.18 | 2.75 | 1.25 | <0.001 |  |  |  |  |  |
| **Fatigue** | 3.14 | 1.52 | 3.60 | 1.44 | 0.005 | 3.22 | 1.49 | 3.95 | 1.47 | <0.001 |  |  |  |  |  |
| **Night’s rest** | 2.81 | 1.51 | 3.36 | 1.59 | 0.001 | 2.90 | 1.53 | 3.83 | 1.57 | <0.001 |  |  |  |  |  |
| **Medication** | 1.13 | 1.24 | 1.58 | 1.63 | 0.009 | 1.19 | 1.34 | 2.03 | 1.74 | <0.001 |  |  |  |  |  |
| **Sexuality** | 1.32 | 1.67 | 1.87 | 1.81 | 0.002 | 1.36 | 1.62 | 2.69 | 2.01 | <0.001 |  |  |  |  |  |
| **Pain** | 3.52 | 1.19 | 3.87 | 1.12 | 0.009 | 3.54 | 1.13 | 4.44 | 1.19 | <0.001 |  |  |  |  |  |
| **Activity avoidance** | 2.44 | 1.51 | 3.35 | 1.46 | <0.001 | 2.67 | 1.49 | 3.73 | 1.62 | <0.001 |  |  |  |  |  |
| **Joint stiffness** | 3.44 | 1.38 | 3.89 | 1.19 | 0.003 | 3.56 | 1.32 | 4.05 | 1.25 | 0.008 |  |  |  |  |  |
